# Downexpression of miR-200c-3p Contributes to Achalasia Disease by Targeting the *PRKG1* Gene

**DOI:** 10.3390/ijms24010668

**Published:** 2022-12-30

**Authors:** Lucia Micale, Carmela Fusco, Grazia Nardella, Orazio Palmieri, Tiziana Latiano, Domenica Gioffreda, Francesca Tavano, Anna Panza, Antonio Merla, Giuseppe Biscaglia, Marco Gentile, Antonello Cuttitta, Marco Castori, Francesco Perri, Anna Latiano

**Affiliations:** 1 Division of Medical Genetics, Fondazione IRCCS Casa Sollievo della Sofferenza, 71013 San Giovanni Rotondo, Italy; 2 Division of Gastroenterology, Fondazione IRCCS Casa Sollievo della Sofferenza, 71013 San Giovanni Rotondo, Italy; 3 Unit of Thoracic Surgery, Fondazione IRCCS Casa Sollievo della Sofferenza, 71013 San Giovanni Rotondo, Italy

**Keywords:** achalasia, in vitro analysis, miR-200c-3p, *PRKG1*, smooth muscle cells

## Abstract

Achalasia is an esophageal smooth muscle motility disorder with unknown pathogenesis. Taking into account our previous results on the downexpression of miR-200c-3p in tissues of patients with achalasia correlated with an increased expression of *PRKG1*, *SULF1*, and *SYDE1* genes, our aim was to explore the unknown biological interaction between these genes and human miR-200c-3p and if this relation could unravel their functional role in the etiology of achalasia. To search for putative miR-200c-3p binding sites in the 3′-UTR of *PRKG1*, *SULF1* and *SYDE1*, a bioinformatics tool was used. To test whether *PRKG1*, *SULF1*, and *SYDE1* are targeted by miR-200c-3p, a dual-luciferase reporter assay and quantitative PCR on HEK293 and fibroblast cell lines were performed. To explore the biological correlation between *PRKG1* and miR-200c-3p, an immunoblot analysis was carried out. The overexpression of miR-200c-3p reduced the luciferase activity in cells transfected with a luciferase reporter containing a fragment of the 3′-UTR regions of *PRKG1*, *SULF1*, and *SYDE1* which included the miR-200c-3p seed sequence. The deletion of the miR-200c-3p seed sequence from the 3′-UTR fragments abrogated this reduction. A negative correlation between miR-200c-3p and *PRKG1*, *SULF1*, and *SYDE1* expression levels was observed. Finally, a reduction of the endogenous level of *PRKG1* in cells overexpressing miR-200c-3p was detected. Our study provides, for the first time, functional evidence about the *PRKG1* gene as a direct target and *SULF1* and *SYDE1* as potential indirect substrates of miR-200c-3p and suggests the involvement of NO/cGMP/PKG signaling in the pathogenesis of achalasia.

## 1. Introduction

Achalasia (MIM#200400) is a rare esophageal motility disorder characterized by aperistalsis of the esophageal body and failure of the lower esophageal sphincter (LES) to relax during swallowing. Though the exact etiology and pathogenesis remain controversial, a body of evidence has emerged showing that idiopathic achalasia is caused primarily by neuronal degeneration of myenteric plexus ganglion cells in the esophagus and LES, possibly as the consequence of a neurotropic virus infection, the effects of a neurotoxin, or myopathy of the smooth muscle cells [1].

MicroRNAs (miRNAs) are small, noncoding RNA molecules that modulate gene expression post-transcriptionally via binding to the 3′ untranslated regions (3′-UTR) of target genes, resulting in mRNA degradation or translation inhibition [2]. miRNAs have been identified as essential mediators in various biological and pathological processes, such as cell proliferation, apoptosis, differentiation, and tumorigenesis [2,3]. Genetic ablation of miRNA machinery as well as loss of deregulation of miRNAs severely compromise immune development and regulation and lead to their involvement in the pathophysiology of immunity, autoimmunity [4], and neurodegenerative disorders [5].

More recently, we profiled miRNAs and miRNA–mRNA regulatory networks in the muscular layers of achalasia patients [6]. In particular, among the target miRNAs of differentially expressed genes associated with the physiological processes significantly enriched in achalasia, we found of interest the indirect association between differentially expressed mRNAs and miR-200c-3p without prior knowledge of the functional interaction [6]. Therefore, we assumed a particular involvement of miR-200c-3p in achalasia pathogenesis. MiR-200c-3p was found to potentially target the protein kinase cGMP-dependent 1 (*PRKG1*), sulfatase 1 (*SULF1*), and synapse defective Rho GTPase Homolog 1 (*SYDE1*) mRNAs involved in smooth muscle contractility and synaptic transmission of cells pathways. Specifically, the downexpression of miR-200c-3p was correlated with an increased expression of *PRKG1*, *SULF1*, and *SYDE1* genes, and even if evident in our study, their interactions are unknown.

In this work, we explored the biological interaction between *PRKG1*, *SULF1*, and *SYDE1* genes and human miR-200c-3p in fibroblast and HEK293 cells by employing a combination of computational and molecular analysis. We hypothesized that miR-200c-3p-mediated *PRKG1* expression could play a crucial role in the etiopathogenesis of achalasia. 

## 2. Results

As shown in Table 1 [6], the Spearman′s correlation analysis demonstrated that *PRKG1*, *SULF1*, and *SYDE1* mRNA levels in the tissues samples of achalasic patients were inversely correlated with miR-200c-3p expression (r = −0.65, r = −0.8558, and −0.7588, respectively). This evidence strongly suggested *PRKG1*, *SULF1*, and *SYDE1* genes as potential targets of miR-200c-3p.

**Figure 1:**
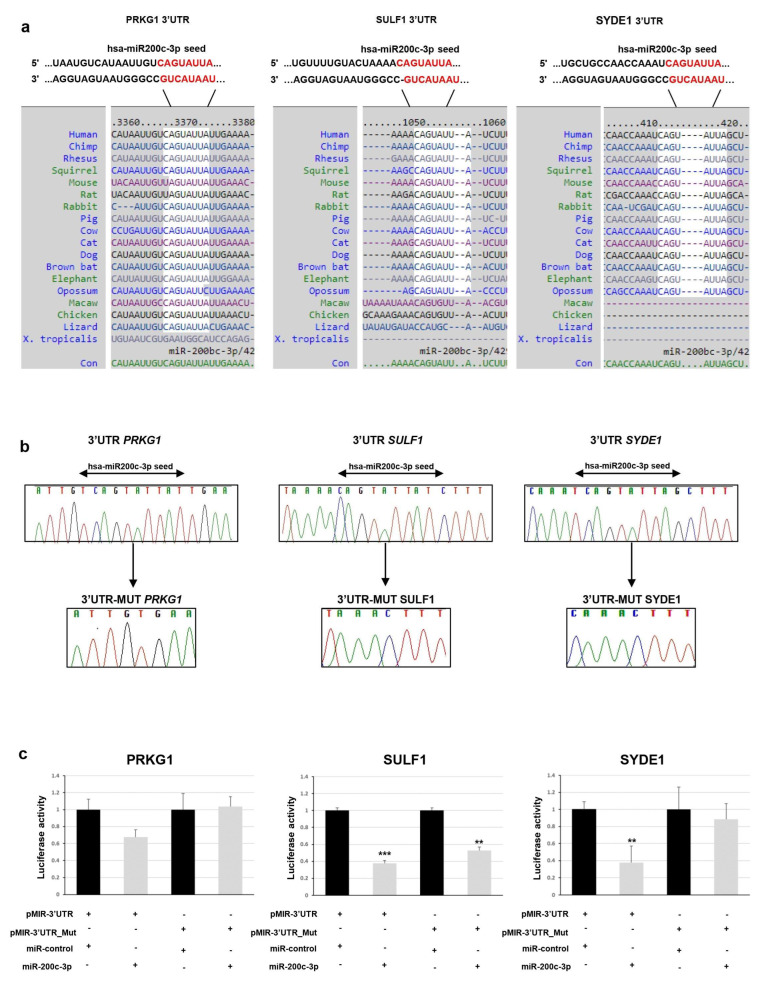
(**a**) Predicted pairing of hsa-miR-200c-3p seed sequence with its target genes is highlighted (upper panel). Positions 3368–3375 of *PRKG1* 3′-UTR, 1052–1059 of *SULF1* 3′-UTR, and 413–420 of *SYDE1* 3′-UTR are highlighted. Seed sequences are highlighted in red. Conserved hsa-miR-200c-3p seed sequence among indicated species is shown. (**b**) Electropherograms showing DNA sequencing analysis of cloned *PRKG1*, *SULF1*, and *SYDE1* 3′-UTR with (upper panel) or not (lower panel) hsa-miR-200c-3p seed sequence. Nucleotide sequences are provided. (**c**) Relative luciferase activity of pmirGLO recombinant vector containing *PRKG1*, *SULF1*, and *SYDE1* 3′-UTR fragments. HEK293 cells were cotransfected with reporter constructs carrying a wild type or miR-200c-3p seed deleted 3′-UTR regions of *PRKG1*, *SULF1*, and *SYDE1* and a synthetic mimic of miR-200c-3p or miR-control. The luciferase activity was normalized with respect to the level of *Renilla* luciferase. Normalized activities of all the 3′-UTR fragments were compared with the empty pmiR vector. Each biological replicate (*n* = 3) was run in three technical replicates. Scale bars represent standard errors (** *p* < 0.05, *** *p* < 0.01).

To determine whether *PRKG1*, *SULF1*, and *SYDE1* genes are really targeted by miR-200c-3p, we first computationally screened the 3′-UTR sequence of these genes to search for putative miRNA binding sites. Bioinformatics analysis indicated a putative conserved binding site of miR-200c-3p in the analyzed region of all three genes. The predicted interaction between miR-200c-3p and its target site in *PRKG1*, *SULF1* and *SYDE1* 3’ -UTR is illustrated in Figure 1a. 

To experimentally test whether *PRKG1*, *SULF1*, and *SYDE1* are directly targeted by miR-200c-3p in vitro, a dual luciferase reporter assay was performed. We cloned a 3′-UTR fragment of *PRKG1*, *SULF1*, and *SYDE1* carrying a wild-type and deleted miR-200c-3p seed sequence (Figure 1b) downstream to a luciferase reporter gene of the pmiR-GLO vector. The luciferase activities driven by these fragments were measured after transient cotransfection of the *PRKG1*, *SULF1*, and *SYDE1* reporter constructs or of the empty vector with a synthetic mimic of miR-200c-3p or miR-control in the HEK293 cell lines. We detected that the overexpression of miR-200c-3p reduced the luciferase activity in the cells containing the 3′-UTR regions of *PRKG1*, *SULF1*, and *SYDE1* by about 35% (*p* = 0.01), 62% (*p* = 0.003), and 62% (*p* = 0.05), respectively, over the negative pmir-GLO empty vector. Conversely, the deletion of the miR-200c-3p seed sequence from the 3′-UTR fragments mediated by in vitro mutagenesis abrogated this reduction in the cells transfected with the reporter constructs containing the 3′-UTR regions of *PRKG1* except for *SULF* and *SYDE1*, which reduced the luciferase activity by about 50% (*p* = 0.02) and 18% (*p* = 0.5), respectively (Figure 1c). 

**Figure 2:**
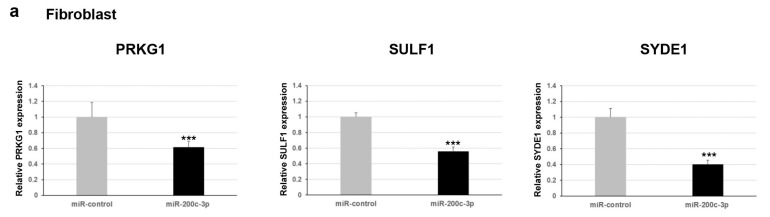

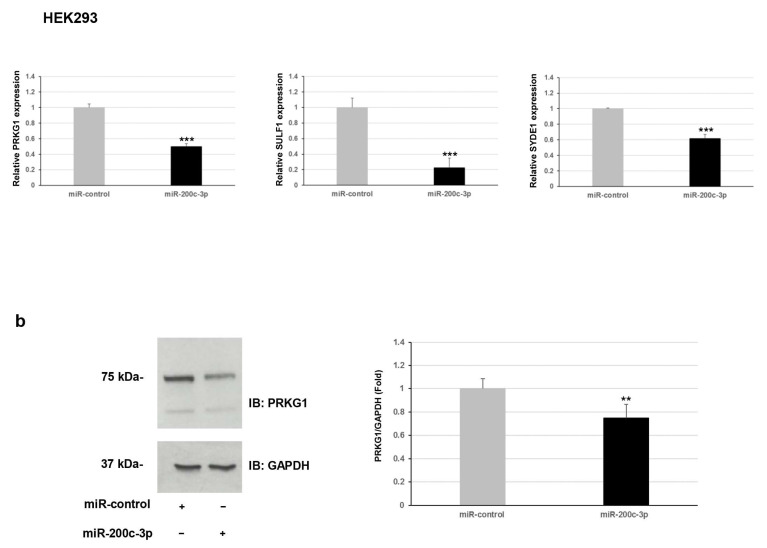
(**a**) Expression profile of *PRKG1*, *SULF1*, and *SYDE1* in fibroblast (upper panel) or HEK293 cells (lower panel) transfected with hsamiR-200c-3p mimic or miR-control by quantitative PCR. Each biological replicate (*n* = 3) was run in three technical replicates. Scale bars represent standard errors (** *p* < 0.05, *** *p* < 0.01). (**b**) Whole protein lysates from HEK293 transfected with hsa-miR200c-3p or miR-control were separated on 10% SDS-gel and subjected to immunoblotting with antiPRKG1 and antiGAPDH antibodies. Levels of PRKG1 and GAPDH were quantified by densitometry using IMAGEJ analysis software. Relative PRKG1 level was normalized compared to GAPDH level. Scale bars represent standard errors (*** *p* < 0.01, biological replicates = 3). Whole protein lysates from HEK293 transfected with.

Furthermore, we explored whether this regulation occurred for endogenous expression levels of *PRKG1*, *SULF1*, and *SYDE1* genes. To verify the effect of miR-200c-3p on the transcript levels of the potential targets, we performed a qPCR analysis on two different types of cells: fibroblast and HEK293. Our results showed a negative correlation between miR-200c-3p and *PRKG1*, *SULF1*, and *SYDE1* expression levels in both fibroblast and HEK293 cell lines transfected with the miR-200c-3p mimic compared to control cells transfected with the miR-control mimic (Figure 2a). Specifically, we observed a decrease of about 40% (*p* = 0.004), 55% (*p* = 0.00006), and 60% (*p* = 0.00004) of endogenous *PRKG1*, *SULF1*, and *SYDE1* transcripts in fibroblast cells transfected with the miR-200c-3p mimic and a reduction of about 50% (*p* = 0.00001), 80% (*p* = 0.0001), and 40% (*p* = 0.009) of endogenous *PRKG1*, *SULF1*, and *SYDE1* transcripts in HEK293 cells exogenously expressing the miR-200c-3p mimic compared to the control cells (Figure 2a and Supplementary Figure S1). Altogether, our luciferase and expression studies suggested *PRKG1* as a direct target and both *SULF1* and *SYDE1* as potential indirect substrates of miR-200c-3p. The use of a specific miR-200c-3p inhibitor did not have any consequence on the luciferase activity and the endogenous *PRKG1*, *SULF1*, and *SYDE1* expression due to undetectable physiological levels of miR-200c-3p in fibroblasts and HEK293 cells. To better explore the biological correlation between *PRKG1* and miR-200c-3p, we performed an immunoblot analysis on protein lysate of HEK293 cells exogenously expressing the miR-200c-3p mimic or miR-control. In keeping with the expression analysis, these assays confirmed a reduction of the endogenous level of PRKG1 in the cells expressing the miR-200c-3p mimic (23%, *p* = 0.0024) compared to the control cells (Figure 2b).

## 3. Discussion

In this study, we identified *PRKG1* as a novel target of human miR-200c-3p. Our in vitro studies on fibroblast and HEK293 cells showed that miR-200c-3p regulates *PRKG1* expression through a direct interaction with the binding site located in the 3′-UTR. 

For this, we have recently established that there is a dysregulated miRNA profile in the esophageal sphincter muscle specimens of patients with achalasia, and we have found three miR-200 family members among the top ten downregulated miRNAs [6]. In particular, miR-200c-3p caught our attention both for the expression level lower than that of the control tissues and for indirect associations with mRNAs without prior knowledge of the interaction. Computational analysis of transcriptomic data identified *PRKG1*, *SULF1*, and *SYDE1* genes as potential targets of miR-200c-3p with an inverse correlation of the expression levels through Spearman’s correlation analysis [6]. In this study, we performed a combination of computational and molecular analysis that suggested *PRKG1* as a direct target and *SULF1* and *SYDE1* as potential indirect substrates of miR-200c-3p. We speculated that the observed indirect interaction between the *SULF1* and *SYDE1* putative target genes and miR-200c-3p could be explained by stating: (i) the miRNA–mRNA interaction could be detected only in specific physiological conditions that are currently unknown, (ii) the interaction is revealed to be very weak, (iii) *SULF1* and *SYDE* are not real targets of miR-200c-3p.

*SULF1* is an integral regulator of essential cell signaling pathways affecting cell growth, proliferation, differentiation, migration [7], and muscle development [8]. *SYDE1* is involved in cytoskeletal remodeling and cell migration, and invasion [9]. *PRKG1* plays a central role in the regulation of cardiovascular and neuronal functions in the contraction and relaxation of smooth muscle tone and the prevention of platelet aggregation [10].

Many examples show that *PRKG1* expression and the contribution to protein kinase production are important to establish contractility of smooth muscle [11,12]. Based on this evidence, we hypothesized that defects in *PRKG1* activity in smooth muscle cells might promote the alteration of contractile function, resembling the pathophysiological aspects of achalasia disease, and the observed inverse correlation between the miR-200c-3p and *PRKG1* (both at transcriptional and translational level) emphasizes its possible involvement in the pathogenesis and development of achalasia. 

Cyclic GMP-dependent protein kinase (*PRKG1*, *PKG*, cGMP-dependent protein kinases) gene encodes type 1 PKG, a serine/threonine-specific protein kinase that acts as a key mediator of the nitric oxide NO/cGMP/cGMP kinase I (cGKI) signaling pathway, including smooth muscle cell relaxation and calcium (Ca^2+^) homeostasis actions [13]. An important pathway regulated by NO in maintaining the vascular tone is NO-soluble guanylate cyclase (sGC)-cyclic guanosine monophosphate (cGMP) signaling. NO stimulates sGC in the vascular smooth muscle cells to induce the formation of cGMP and causes stimulation of cGMP-dependent protein kinases (cGKs: cGKI and cGKII), which in turn elicit voltage-dependent ion channels and mediate the physiological functions of cGMP. cGKs inhibit Ca^2+^ release from the endoplasmic reticulum through the IRAG substrate and alternatively activate myosin-light-chain phosphatase by inhibiting the MLC kinases, with both mechanisms resulting in smooth muscle relaxation [14]. Moreover, proteins that are phosphorylated by cGKI regulate platelet activation and adhesion, cardiac function, intestinal and neuronal functions, gene transcription, and modulate cell growth [13]. cGKI has been detected at high concentrations in all types of smooth muscle cells where increased cGMP and PRKG1 activity affects the expression of smooth muscle-specific contractile proteins [11], and restoration of the expression of *PRKG1* reestablishes contractile function in cultured vascular smooth muscle cells [12]. Collectively, this information indicates that *PRKG1* expression and contribution to protein kinase production are important to establish contractility in smooth muscle.

It has been demonstrated that NO and vasoactive intestinal peptide (VIP) induce smooth muscle relaxation by inhibitory neurons [15], and excitatory neurons induce Ca^2+^ ion activity and contract smooth muscles by releasing acetylcholine in the esophagus [16]. In patients with achalasia, the LES displays a prominent NO-dependent hyperpolarization of resting membrane potential and relaxation in response to the activation of enteric inhibitory neurons. Worth nothing, the nerves that are destroyed or defective in achalasia seem to be the principal motor nerves of the esophageal smooth muscle, nerves that act on the muscle by releasing NO [17]. Finally, lower expression of Ca^2+^ storage proteins was detected in LES in patients with achalasia and led to speculation that this reduced expression might be accompanied by increased intracellular free Ca^2+^ followed by enhanced contractility and elevated LES pressure [18]. 

Currently, little is known about the role of miRNAs in achalasia [6,19,20,21,22]. miR-200c-3p belongs to the miR-200 family, which includes miR-200c and miR-141 located on chromosome 12p13 and miR-200a/b and miR-429 on chromosome 1p36. Deregulation of miR-200c has been shown in several cancer types, such as breast, colorectal, endometrial, gastric cancer, hepatocellular tumor, lung, oral squamous cell carcinoma, ovarian, and pancreatic [23]. miR-200c has been initially shown to regulate epithelial–mesenchymal transition; afterwards, it has been demonstrated that it has versatile roles in proliferation, cell cycle control, apoptosis, invasion, drug resistance, oxidative stress [24,25], and neuronal cell death [26]. 

This functional study provides evidence that miR-200c-3p directly regulates *PRKG1* and suggests the involvement of NO/cGMP/PKG signaling in the pathogenesis of achalasia. miR-200c-3p-mediated *PRKG1* regulation could induce an achalasia-like phenotype in the smooth muscle cells through suppression of the NO/cGMP pathway. Rescuing consistent levels of protein kinase could have several benefits, such as decreasing the contractile state of smooth muscle. Our study has some limitations. First, the evaluation of the molecular mechanism of miR-200c-3p-mediated *PRKG1* in vivo and second, the pathogenic effect of miR-200c-3p on achalasia. Future studies are needed to confirm our findings in order to identify the therapeutic and prognostic potential of miR-200c-3p in achalasia.

## 4. Materials and Methods

### 4.1. Computational Analysis

TargetScan was used to search for predicting miR-200c-3p direct or indirect targets in mammals (https://www.targetscan.org/vert_80/; Release 8.0: September 2021).

### 4.2. pmir-GLO Constructs

Fragments of 1011bp, 1156bp, and 968bp encompassing the miR-200c-3p binding site located in the 3′-UTR of *SULF1*, *PRKG1*, and *SIDE* genes, respectively, were amplified by using Taq polymerase Pfu (Promega, Madison, WI, USA). Primers for PCR were designed according to the 3’-UTR genomic sequence of human *SULF1*, *PRKG1*, and *SIDE* genes (Table S1). After PCR amplification, PCR products were purified and subjected to restriction enzyme digestion with XhoI (New England BioLabs, Ipswich, MA, USA) and XbaI (New England BioLabs, Ipswich, MA, USA) and then cloned into the pmir-GLO dual-luciferase miRNA target expression reporter vector (Promega, Madison, WI, USA).

In vitro mutagenesis was employed to delete the miR-200c-3p binding site from pmir-SULF1, pmir-PRKG1, and pmir-SIDE by using the QuickChange II site-directed mutagenesis kit (Stratagene, Santa Clara, CA, USA) according to the manufacturer’s instructions. All constructs containing wild-type and mutated fragments were verified by Sanger sequencing using BigDye Terminator v1.1 sequencing kit (Thermo Fisher Scientific, Waltham, MA, USA), purified using DyeEx plates (Qiagen, Hilden, Germany), and resolved on ABI Prism 3130 Genetic Analyzer (Thermo Fisher Scientific, Waltham, MA, USA). Sequences were analyzed using the Sequencer software (Gene Codes, Ann Arbor, MI, USA). 

### 4.3. Cell Cultures

Established primary dermal fibroblasts from healthy human donors were cultured in Dulbecco’s Modified Eagle Medium/Nutrient Mixture F12 (D-MEM-F12) (Thermo Fisher Scientific, Waltham, MA, USA) plus 10% fetal bovine serum (FBS) (Thermo Fisher Scientific, Waltham, MA, USA) and 1% penicillin and streptomycin (P/S, 100 U/mL and 100 μg/mL, respectively) (Thermo Fisher Scientific, Waltham, MA, USA). Human embryonic kidney (HEK) 293 cell lines were maintained in D-MEM with Glutamax supplemented with 10% FBS and 1% P/S. Cells were grown in a 5% CO_2_ incubator at 37 °C. The cells were plated and grown at about 90% confluence before transfection.

### 4.4. Dual-Luciferase Reporter Assay

HEK293 cells were seeded at density of 1.0 × 10^5^ cells/mL per well in a 12-well plate and then transfected with 150 ng of pmir-GLO constructs together with 5 pmol of miR-200c-3p mimic (Catalogue #: MSY0000437, Qiagen, Hilden, Germany) or 5 pmol of miRNA mimic control (miR-control, catalog #: MSY0000416, Qiagen, Hilden, Germany) using 1 ul of Lipofectamine 2000 (Thermo Fisher Scientific, Waltham, MA, USA) according to manufacturer’s instructions. Forty-eight hours after transfection, cells were lysed in the passive buffer and assayed for both firefly and Renilla luciferase activities using the Dual Luciferase Assay System (Promega, Madison, WI, USA) in a Glomax 96-microplate luminometer (Promega, Madison, WI, USA). Firefly luciferase activity was normalized to Renilla luciferase activity for each transfected well. Normalized firefly luciferase activity for each construct was compared to that of the pmir-GLO empty vector. Values are the mean ± standard error of the mean of three experimental replicates from three independent transfections. 

### 4.5. miRNA Transfection

Primary dermal fibroblasts and HEK293 cells were seeded at density of 2.0 × 10^5^ cells/mL per well in a 6-well plate and then transfected with 25 pmol of synthetic hsa-miR-200c-3p mimic or hsa-miR-200c-3p inhibitor (Catalogue #: MIN0000617, Qiagen, Hilden, Germany) using 5 μL of Lipofectamine 3000 (Thermo Fisher Scientific, Waltham, MA, USA) following the manufacturer’s instructions. The miR-control mimic and inhibitor were used as miRNA control at equimolar concentrations. Triplicates were used in each cell experiment. Forty-eight hours after transfection, the cells were harvested and then subjected to RNA extraction.

### 4.6. RNA Extraction and Retrotranscription

Total RNA, including smaller microRNAs, was extracted from HEK293 and primary dermal fibroblast cell lines using the miRNeasy Mini Kit (Qiagen, Hilden, Germany) according to the manufacturer’s instructions. Subsequently, the RNA was purified, eluted, and collected. RNA quality was evaluated on the Agilent 2200 Tape Station System 2200 (Agilent Technologies, Palo Alto, CA, USA) using RNA ScreenTape Assays (Agilent Technologies, Palo Alto, CA, USA), and RNAs with an RNA integrity number (RIN) ≥7.0 were retained for the subsequent RT-PCR analysis. RNA concentrations were estimated by ND-1000 Spectrophotometer (NanoDrop Technologies, Wilmington, DE, USA). For *SYDE1*, *SULF1*, and *PRKG1* expression detection, a mixture containing 0.1 μg of total RNA from HEK293 and fibroblast cell lines was reverse transcribed for 10 min at 25 °C and 2 h at 37 °C using the High-Capacity cDNA Reverse Transcription Kit (Thermo Fisher Scientific, Waltham, MA, USA) and random hexanucleotides. For miR-200c-3p detection, 10 ng of total RNA was reverse transcribed using the Taq-Man MicroRNA Reverse Transcription kit (Thermo Fisher Scientific, Waltham, MA, USA) in accordance with the manufacturer’s instructions. The miR-200c-3p 5X primers and the endogenous control RNU6B 5X primers (Assay ID: 000505 and Assay ID: 001093, respectively; Thermo Fisher Scientific, Waltham, MA, USA) were included in the reaction.

### 4.7. Real-Time Quantitative PCR Analysis of PRKG1, SULF1, and SYDE1 mRNA Levels and miR-200c-3p

Specific oligos for *PRKG1*, *SULF1*, and *SYDE* were designed using the Primer Express program [27]. Primers were checked both by Basic Local Alignment Search Tool (BLAST) and BLAST-like alignment tool (BLAT) against the human genome to ensure specificity. Primers are listed in Table S1. Reactions were run in triplicate in 10 µL of final volume with 10 ng of sample cDNA, 0.3 mM of each primer, and 1x Power SYBR Green PCR Master Mix (Thermo Fisher Scientific, Waltham, MA, USA). Reactions were set up in a 384-well plate format and run in an ABI Prism7900HT (Thermo Fisher Scientific-Applied Biosystems, Carlsbad, CA, USA). Raw cycle threshold (Ct) values were obtained using SDS 2.4 (Applied Biosystems, Carlsbad, CA, USA). Calculations were carried out by the comparative Ct method as reported in Livak, K.J. [28] Significance was determined by a two-tailed unpaired t-test for means. *GAPDH* and *18S* genes were used as references for “Ct value” normalization. 

Quantitative PCR analysis of miR-200c-3p was performed on transfected cells using the TaqMan microRNA Assay (Applied Biosystems, Foster City, CA, USA) on ABI Prism7900HT in accordance with the manufacturer’s instructions. Briefly, a 20 μL PCR reaction including 2.5 μL of RT product, 1X TaqMan Universal PCR Master Mix, and 1X of the corresponding TaqMan Gene Expression Assay (miR-200c-3p 20X, Assay ID: 000505; RNU6B 20X, Assay ID: 001093, Thermo Fisher Scientific, Waltham, MA, USA) was incubated in 96-well plates at 95 °C for 10 min followed by 40 cycles of 95 °C for 15 sec and 60 °C for 1 min. PCR reactions were performed in triplicate. Relative quantities of each cDNA were calculated using the ΔΔCt method after normalization with endogenous reference RNU6B.

### 4.8. Western Blotting and Quantification of Protein Level

HEK293 cells were seeded at density of 2.0 × 10^5^ cells/mL per well in a 6-well plate and then transfected with miR-200c-3p mimic or miR-CTRL using Lipofectamine 2000 and after 72 h, analyzed for Western blotting assay. Cells were lysed in 1X Dulbecco’s Phosphate-Buffered Saline (D-PBS), 0.025% NP-40, protease inhibitors (Roche, Pasadena, CA, USA), and phospho-inhibitors (Roche, Pasadena, CA, USA). Supernatant was cleared by centrifugation. Proteins were resolved by electrophoresis on 10% SDS-gel followed by transfer to nitrocellulose membrane. Membranes were probed with antiPRKG1 (1:500, #3248, Cell Signaling, Dellaertweg, The Netherlands) and antiGAPDH (1:1000, sc-47724 Santa Cruz, CA, USA) antibodies. Detection was achieved using horseradish peroxidase-conjugated antimouse (1:10000, 1706516, BioRad, Hercules, CA, USA) and antirabbit (1:10000, #1706515, BioRad, Hercules, CA, USA) antibodies. Signals were acquired using Amersham Hyperfilm ECL (GE Healthcare, Little Chalfont, UK) chemiluminescence system. To analyze the PRKG1 protein level, the relative band intensity was quantified using Image J software, and the ratio of PRKG1 to GAPDH level was calculated. 

### 4.9. Statistical Methods

Statistical analysis was performed using unpaired, two-tailed Student’s *t*-test, and *p*-value ≤ 0.05 was considered significant.

## Figures and Tables

**Table 1:** Indirect associations between *PRKG1*, *SULF1*, and *SYDE1* genes and differentially expressed miR-200c-3p without prior knowledge of the interaction.

miRNA	*p-Value*	*Fold Change*	*Target Gene*	*p-Value*	*Fold Change*	Spearman Correlation Coefficient	*p-Value* Spearman Correlation Coefficient	Associated Function
hsa-miR-200c-3p	9.11 × 10^−15^	−2724.21	SULF1	3.01 × 10^−6^	4.16688	−0.855882	2.34 × 10^−5^	Contractility of smooth muscle
hsa-miR-200c-3p	9.11 × 10^−15^	−2724.21	PRKG1	3.25 × 10^−11^	4.99205	−0.65	0.00641571	Contractility of smooth muscle
hsa-miR-200c-3p	9.11 × 10^−15^	−2724.21	SYDE1	3.01 × 10^−6^	2.80904	−0.758824	0.00065403	Synaptic transmission of cells

## Supplementary Materials

The following supporting information can be downloaded at: https://www.mdpi.com/article/10.3390/ijms24010668/s1.
