# Supplementary material for: Downexpression of miR-200c-3p Contributes to Achalasia Disease by Targeting the PRKG1 Gene
Source: Int J Mol Sci. 2022 Dec 30;24(1):668. doi: 10.3390/ijms24010668 (PMC9820813; doi:10.3390/ijms24010668)
Supplement: Supplementary file 1 [file ijms-24-00668-s001.zip › ijms-2094132-supplementary.pdf]

**Supplementary Figure S1.** Expression profile of hsa-miR-200c-3p in both fibroblast and HEK293 cells transfected with miR-200c-3p mimic and miR-CNT mimic by quantitative PCR.

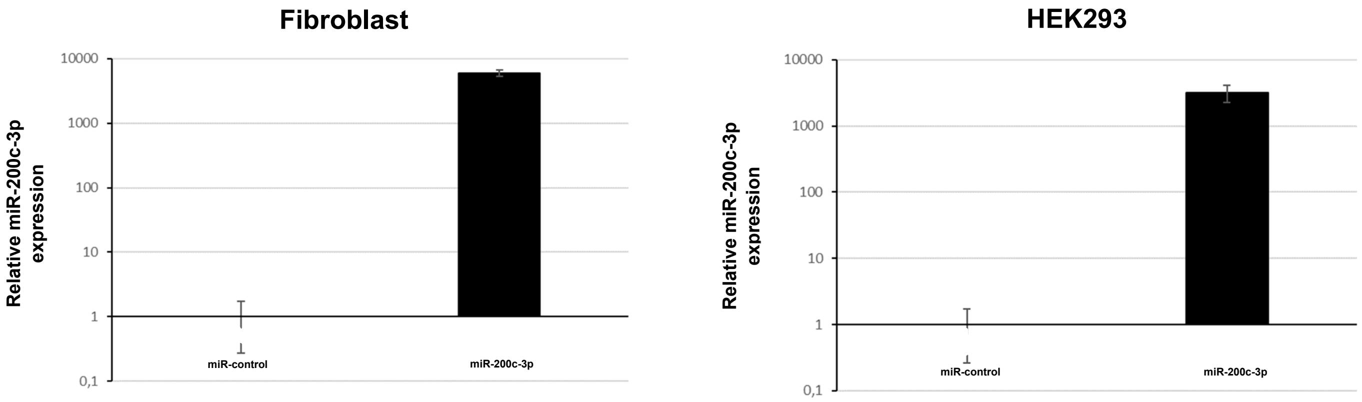

**Table S1. Sequences of primers used in this study.**

| Gene | Sequence | Use |
|---|---|---|
| PRKG1-3'UTR-(XhoI)-F | CGCGCGctcgagCAGGAAAACTGAAACTGCCAAGG | Cloning |
| PRKG1-3'UTR-(XbaI)-R | CGCGCGtctagaGCATCCTGTTTTGTTGAGAGGTGC | Cloning |
| SYDE1-3'UTR-(XhoI)-F | CGCGCGctcgagGACCCCGGTTAGTAAGGACC | Cloning |
| SYDE1-3'UTR-(XbaI)-R | CGCGCGtctagaCCACAAAGATGGAGGTGCGGAAAAG | Cloning |
| SULF1-3'UTR-(XhoI)-F | CGCGCGctcgagCACCGAAGTAATTCCAGCATAGC | Cloning |
| SULF1-3'UTR-(XbaI)-R | CGCGCGtctagaTAAGCTTCTTAGTCATGCCAAGA | Cloning |
| PRKG1-3'UTR-MUT-F | TAGACTTGAATTAATGTCATAATTGTGAAAATTATGTCAACTGTACTGTT | Mutagenesis |
| PRKG1-3'UTR-MUT-R | AACAGTACAGTTGACATAATTTTCACAATTATGACATTAATTCAAGTCTA | Mutagenesis |
| SYDE1-3'UTR-MUT-F | GGCCCTCTTGCTGCTGCCAACCAAACTTTGAGCACTGCACTGTTTCTCCC | Mutagenesis |
| SYDE1-3'UTR-MUT-R | GGGAGAAACAGTGCAGTGCTCAAAGTTTGGTTGGCAGCAGCAAGAGGGCC | Mutagenesis |
| SULF1-3'UTR-MUT-F | GCTTGTTTGTTTGTTTTGTACTAAACTTTTGAATATCGTAGGGACATAAG | Mutagenesis |
| SULF1-3'UTR-MUT-R | CTTATGTCCCTACGATATTCAAAAGTTTAGTACAAAACAAACAAACAAGC | Mutagenesis |
| PRKG1-RT-F1 | CGCAGACGTACAGGTCCTT | qPCR |
| PRKG1-RT-R1 | ATCCTTGGACCTTTCGGACTTG | qPCR |
| SYDE-RT-F1 | TGCAGCGGTCTGCCTATCT | qPCR |
| SYDE-RT-R1 | GAGGATGCCAGTGATGACATTG | qPCR |
| SULF1-RT-F1 | CCAGCAGAAGCCAAAGAAAGAG | qPCR |
| SULF1-RT-R1 | TGTACTTTGGAGTCCCCTGGTTT | qPCR |
| GAPDH-RT-F | AATCCCATCACCATCTTCCA | qPCR |
| GAPDH-RT-R | AAATGAGCCCCAGCCTTC | qPCR |
| 18S-RT-F | CCCAGTAAGTGCGGGTCATAA | qPCR |
| 18S-RT-R | ACGGGCGGTGTGTACAAAG | qPCR |
